# Design-related risk factors for revision of primary cemented stems: Analysis of 3 common stems in the Swedish Hip Arthroplasty Register

**DOI:** 10.3109/17453674.2010.501739

**Published:** 2010-07-16

**Authors:** Truike M Thien, Johan Kärrholm

**Affiliations:** Department of Orthopaedics, Institute of Clinical Sciences at Sahlgrenska Academy, University of GothenburgSweden

## Abstract

**Background and purpose:**

Even small design variables of the femoral stem may influence the outcome of a hip arthroplasty. We investigated whether design-related factors play any role in the risk of non-aseptic revision of the 3 most frequently used primary cemented stem designs in the Swedish Hip Arthroplasty Register.

**Patients and methods:**

We studied 71,184 primary cemented femoral stem implants (21,008 Exeter polished stems, 43,036 Lubinus SPII stems, and 7,140 Spectron EF Primary stems) that were inserted from 1999 through 2006. Design-specific characteristics were analyzed using separate Cox regression models that were adjusted for sex, age, diagnosis, incision, and number of operations (first vs. second).

**Results:**

The crude revision rate varied between 0.8% (Lubinus SPII) and 1.4% (Spectron Primary). For the Exeter stem, the smallest femoral head diameter (22 mm) was associated with a higher risk of revision. No other design-specific parameters influenced the risk of revision of the Exeter stem. The smallest Lubinus stem size, a stem with extended neck length combined with a femoral head with increasing neck length, or the use of a cobalt-chromium head had a negative influence on the outcome. For the Spectron stem, the risk of revision was elevated for the smallest stem and for increasing offset calculated as the combined effect of high offset design and increasing neck length.

**Interpretation:**

Overall revision rates were low, but for two of the stems studied design factors such as size and neck length or offset influenced the risk of non-aseptic revision.

## Introduction

The Swedish Hip Arthroplasty Register has proved to be an important tool for continuous monitoring of outcome after total hip replacement (THR) in Sweden (Herberts and Malchau 1997, 2000). The registry covers all operating units. Data are reported annually to the participating clinics and are available to the public on the internet. More than 14,000 primary THRs were performed in Sweden in 2008 and the number is increasing every year. In 2008, about 74% were all-cemented.

Small changes in femoral stem design can lead to catastrophic deterioration of clinical outcome in THR, e.g. the matte surface on the Exeter design (Rockborn et al. 1993, Middleton et al. 1998), the rough surface on the VerSys femoral stem (Della Valle et al. 2005), and the rough surface on the Iowa Hip (Mohler et al. 1995, Sporer et al. 1999). Also, other variations in standard stem designs such as stem size, neck angle (CCD), and offset may influence the behavior of stems in THR and have an influence on the risk of failure (Olofsson et al. 2006).

Since 1999, the Swedish Hip Arthroplasty Register has increased the capture of data to also embrace the individual article number of each component in an individual THR. This means that information such as stem size, neck length, head size, and material used in the individual THR is available.

We investigated whether these design-related factors play any role in the risk of non-aseptic revision of the femoral component in primary cemented THR, when adjusted for bias caused by demographic and surgery-related factors recorded in the Register. To obtain sufficiently large variation in each design studied, we restricted this analysis to the three stem designs that were used most frequently between 1999 and 2006.

## Materials and methods

From 1999 through 2006, 100,786 femoral implants were inserted. Of these, 1,702 underwent a first-time aseptic revision during the same period. We used only revision of the femoral component with or without any simultaneous revision of the acetabular component as endpoint.

The 3 stem designs studied were Exeter polished (n = 22,577), Lubinus SPII (n = 44,904), and Spectron EF Primary (n = 7,361). Specially designed implants (e.g. some dysplasia or extra-long stems) were excluded, as were femoral components used in small numbers (n < 50). All THR revised due to infection, those performed because of tumors, and cases with missing data were excluded. These exclusions left 71,184 cemented femoral stem implants (21,008 Exeter polished stems, 43,036 Lubinus SPII stems, and 7,140 Spectron EF Primary stems).

The article number of each component was recoded into classified variables. Stem size, neck length, and offset were recorded for all designs. An offset variable including the presence of offset version and neck length was constructed (Lubinus SP2, Spectron EF Primary). Head material could be studied in 2 designs (Exeter, Lubinus SP2) and femoral head size (Exeter), type of taper (Exeter), and CCD angle (Lubinus SP2) in one design each (Tables 1–3).

**Table 1.** Implant-related parameters for the polished Exeter stem entered into the statistical analysis

Classification/Name	Number
*Stem*	
Size	
light weight–0	2,781
light weight–1	8,019
narrow	6,205
standard	2,860
heavy duty	1,013
ultra heavy duty	130
Offset (mm)	
37.5	4,951
44	15,416
50	641
Taper	
old	5,775
V40	15,233
*Head*	
Material	
standard	5,751
alumina ceramic	630
Orthinox	14,627
Length	
short	3,707
medium	14,671
long	2,542
x-long	88
Diameter (mm)	
22	1,567
26	443
28	18,750
30	248

**Table 2.** Implant-related parameters for the Lubinus SPII stem entered into the statistical analysis. All implants were supplied with 28-mm heads

Classification/Name	Number
*Stem*	
Size	
x-small	4,445
small	11,158
medium	14,187
large	9,350
x and xx-large	3,896
CCD	
117	1,388
126	36,179
135	5,469
Offset	
standard (s-off)	41,276
x-offset (x-off)	1,760
*Head*	
Material	
standard	36,802
alumina ceramic	6,234
Length	
short (s, 46)	12,538
medium (m, 49)	19,797
long (l, 53)	9,833
x-long (xl, 60)	868
Offset + neck ^**a**^	
1: s-off + s neck	11,727
2: s-off + m neck or x-off + s neck	19,921
3: s-off + l neck or x-off + m neck	10,290
4: s-off + xl neck or x-off + l neck or xl neck	1,098

^**a**^ Neck length as extracted from the database after assembly of the stem, coded into 4 classes. A higher number means a longer neck. Class 4 included 32 implants with combined x-off and xl-neck.

**Table 3.** Implant-related parameters for the Spectron EF Primary stem entered into the statistical analysis. All implants were supplied with 28-mm heads made of cobalt-chromium alloy

Classification/Name	Number
*Stem*	
Size	
1	1,101
2	3,501
3	1,987
4+5	551
Offset	
standard (s-off)	4,976
h-offset (h-off)	2,164
*Head*	
Length	
short or standard (s, -3, 0)	3,847
long (l, +4)	2,264
x-long (xl, +8)	822
x-long+ (xxl, +12 or xxxl, +16)	207
Offset + neck ^**a**^	
1: s-off or h-off + s neck	2,696
2: s-off + l neck or h-off + s neck	2,716
3: s-off + xl neck or h-off + l neck	1,275
4: s-off + xx l neck or h-off + xl neck	362
5: s-off + xxxl neck or h-off + xxl neck or xxxl neck	91

^**a**^ Neck length as extracted from the database after assembly of the stem, coded into 5 classes. A higher number means a longer neck.

### Femoral stem components

#### Exeter polished

The highly polished tapered collarless Exeter prosthesis (Stryker/Howmedica/Osteonics, Allendale, NJ) is made of low-corrosion stainless steel (Orthinox) and is equipped with a hollow centralizer that is made of pre-polymerized acrylic cement and applied to the tip of the stem to allow subsidence into the centralizer. Its Ra is about 0.1–0.3 mm. The polished Exeter stem has so far been analyzed as a uniform design in the Swedish Hip Arthroplasty Register. However, according to the product lists from the manufacturer, the stem designs used in Sweden between 1999 and 2006 consisted of 2 slightly different prosthesis systems, the older Exeter and the newer Exeter V40. We investigated these designs both individually and together as one group.

The shape of the part of the stem in contact with the cement is equal for both stem designs, but the V40 design has a spigot at the head-neck junction. The stem is available in 7 sizes, with 6 increasing offsets. Different neck lengths with the modular heads provide further offset options.

#### Lubinus SPII

The Lubinus SPII stem prosthesis (Lubinus eccentric; Waldemar Link, Hamburg, Germany) without a centralizer is made of cobalt-chromium alloy, is double curved with anterior and posterior ridges, and has an anatomic shape and a matte surface (Ra = 1.5 μm). The stem is available in 7 increasing sizes, each with a standard or extended neck length, and with 3 neck angles (117°, 126°, and 135°). The analysis was restricted to the most commonly used stem length (150 mm).

#### Spectron EF Primary

The straight-stem Spectron EF Primary prosthesis (Spectron Primary; Smith and Nephew, Memphis, TN) is made of cobalt-chromium alloy (CoCr). The proximal one-third of the stem is grit-blasted with a surface roughness (Ra) of 2.8 μm and the distal part is smoother with Ra = 0.7 μm. The stem has a centralizer and is available in 5 sizes with increasing length and thickness with normal or high offset. All sizes have the same neck angle (131°). In the high-offset design, the neck is displaced medially and the neck becomes longer for one and the same head taper.

### Follow-up

The average follow-up period for all the femoral stems analyzed was 3.5 (SD 2.2) years: 3.3 (SD 2.1) years for the Exeter, 3.3 (SD 2.2) years for the Lubinus SPII, and 3.6 (SD 2.2) years for the Spectron EF Primary.

### Statistics

SPSS version 16.0 for Windows was used. Design-specific characteristics were analyzed using separate Cox regression models that were adjusted for sex, age, diagnosis, incision, and number of operation (first or second). The operation was classified as the second operation when the patient had been operated earlier with a total hip arthroplasty on the contralateral side. The assumption of proportional hazards was verified by hazard function plots that showed a reasonable proportionality over time. For the time intervals chosen, no crossing or clearly deviating survival of the design parameters was observed. In the Exeter group, the V40 taper had only been in use during the later part of the period (mean follow-up: 2.4 years; mean follow-up for old taper: 5.4 years). In addition, 22-mm heads for the Exeter stem were mainly used during the later part of the observation period. We therefore decided to restrict the Cox regression analysis of the Exeter stem and only include aseptic revisions up to 3 years. In the Lubinus SPII and Spectron Primary groups, most stems were inserted with 28-mm heads. Thus, implants with other head sizes were excluded. All design parameters were classified using the group with highest number of observations as reference (Tables 1–3). Adjusted risk ratios, 95% confidence intervals (CIs), and p-values are reported. A p-value < 0.05 was considered to be significant.

## Results

### The Exeter stem (Table 1)

Although the Exeter stem is available in 7 sizes with 6 increasing offsets, the largest stem sizes and offsets were used in insufficient numbers (n < 50) and we therefore analyzed them together as one class for the 2 largest stem sizes and as one class for the 2 largest offsets, resulting in 4 classes of offset and 5 classes of size. For the older version of the Exeter stem and the newer Exeter V40 stem, the average follow-up was 5.4 (SD 1.9) years and 2.4 (SD 1.5) years, respectively.

When the stem design factor (“old” or V40) was disregarded, the risk of revision was only higher for 22-mm femoral heads compared to 28-mm heads. None of the other implant-related variables influenced the risk of stem revision (Table 4). However, the “old” Exeter type was revised most frequently because of aseptic loosening and the V40 type was revised most frequently due to dislocation (Table 5).

**Table 4.** Relative risks of aseptic stem revision during entire observation period (Lubinus SP, Spectron EF Primary) or within 3 years (Exeter) and 95% CI for implant-related parameters for each stem type. Data from Cox regression adjusted for age, sex, diagnosis, bilaterality, side, and implant-specific parameters (see text). Implant-related parameters with statistically significant influence are shown

	Relative Risk	95% CI		p-value
	[Exp (B)]	Lower	Upper	
**Exeter polished** (n = 21,008)				
* Femoral head diameter*				
22 mm	2.37	1.32	4.23	0.004
26 mm	0.02	0.00	30.03	0.3
28 mm, reference	1.00			
30 mm	0.79	0.19	3.28	0.7
**Lubinus SPII** (n = 43,036)				
* Size*				
x-small	1.72	1.22	2.44	0.002
small	0.99	0.74	1.32	0.9
medium, reference	1.00			
large	0.94	0.70	1.26	0.7
x and xx-large	1.01	0.69	1.47	1.0
* Head*				
ceramic	0.52	0.36	0.76	0.001
cobalt-chromium, reference	1.00			
* Offset + neck*				
1	0.73	0.55	0.97	0.03
2 reference	1.00			
3	1.11	0.86	1.43	0.4
4	1.72	1.09	2.71	0.02
**Spectron EF Primary** (n = 7,140)				
* Size*				
1	2.23	1.25	3.97	0.006
2 reference	1.00			
3	0.75	0.40	1.41	0.4
4+5	0.55	0.17	1.84	0.3
* Off-set + neck*				
1	1.27	0.68	2.37	0.4
2 reference	1.00			
3	1.54	0.80	2.98	0.2
4	2.03	0.81	5.11	0.1
5	6.89	2.54	18.63	< 0.001

**Table 5.** Revisions for all the femoral stems analyzed (infections excluded)

	Exeter Polished (All)	Exeter (old)	Exeter V40	Lubinus SPII	Spectron EF
Primary					
	% (n)	% (n)	% (n)	% (n)	% (n)
Aseptic Loosening	0.4 (96)	1.1 (71)	0.2 (25)	0.3 (114)	0.8 (57)
Dislocation	0.5 (116)	0.6 (39)	0.5 (77)	0.5 (244)	0.5 (35)
Fracture	0.2 (43)	0.3 (18)	0.2 (25)	0.0 (17)	0.0 (3)
Implant failure	0.0 (3)	0.0 (0)	0.0 (3)	0.0 (8)	0.0 (0)
Technical reason	0.1 (12)	0.1 (7)	0.0 (5)	0.0 (12)	0.1 (4)
Pain only	0.0 (3)	0.0 (0)	0.0 (3)	0.0 (5)	0.0 (2)
Total	1.2 (273)	2.1 (135)	1.1 (138)	0.8 (400)	1.4 (101)

### The Lubinus SPII stem (Table 2)

The largest 2 stem sizes were used in less than 50 cases and they were therefore analyzed together as one class, resulting in 6 classes of stem size. Only head diameters of 28 mm were included because too few other head sizes were used.

Only the smallest stem size (extra-small) was associated with an increased risk of stem revision compared to all other sizes (Table 4). The risk of revision in the short term was almost half for ceramic heads when compared to the group with cobalt-chromium heads (RR = 0.52; p = 0.001) (Table 4). The reasons for revision of both the ceramic and the cobalt-chromium heads are shown in Table 6. The risk of aseptic revision was reduced in cases with a constructed offset variable corresponding to a short neck length (RR = 0.73; p = 0.3) and increased with use of the longest neck length (RR = 1.72; p = 0.02) (Table 4).

**Table 6.** Number of revisions and causes of revision for Lubinus SPII stems with either cobalt-chromium or aluminium oxide femoral heads (infections excluded)

	Cobalt-chromium % (n)	Aluminium oxide % (n)
Aseptic loosening	29 (104)	24 (9)
Dislocation	61 (222)	53 (20)
Fracture	4.4 (16)	3 (1)
Implant failure	1.1 (4)	11 (4)
Technical reason	2.5 (9)	8 (3)
Pain only	1.1 (4)	3 (1)
Other mechanical reason	1.1 (4)	0 (0)
Total	100 (363)	100 (38)

### The Spectron implant (Table 3)

Only cobalt-chromium heads with a head diameter of 28 mm were included because the heads made of other materials and with other diameters were used in insufficient numbers (n < 50). The risk of revision was higher for the smallest stem (RR = 2.2; p = 0.006) (Table 4). The risk of aseptic revision was almost 7 times higher in the group with the longest assembled neck lengths based on stem offset and neck length (RR = 6.9; p < 0.001) (Table 4).

## Discussion

Overall, the early revision rate for the 3 most frequently used cemented THRs in Sweden is low (Table 5). Thus, extensive material is required for reliable analysis. The advantage of this study is that it is representative of a wide spectrum of orthopedic surgeons with variable clinical experience, and that it covers a whole nation with complete coverage of all hospitals and very few dropouts (about 1–2% of individual operations annually) (Kärrholm et al. 2008).

We have already performed an analysis similar to the present one (Kärrholm and Herberts 2006). At that time, the follow-up was shorter and all types of aseptic revisions were included—also isolated revisions of the cup. The present analysis is based only on stem revision as the primary outcome variable.

One problem with our analysis is the short follow-up, which is related to the start of recording of article numbers in 1999. The registry is continuously updated with modifications of implants, and we can therefore directly relate any change in the survival of an implant to any modification of that implant design. However, changes in survival in the short term must be interpreted with care, especially as the increased risk of revision turned out to be due to recurrent dislocation, as dislocation is often an early complication (Woolson and Rahimtoola 1999, Sanchez-Sotelo and Berry 2001) and might have nothing to do with the specific modification of the implant design. Sometimes a longer follow-up time is necessary in order to identify substantial changes in survival outcome.

In addition, the definition of failure in the registry—revision with exchange or extraction of at least one part of the THR—is a problem and a limitation in this study. The cohort of unrevised patients with problems is probably as large as the revised cohort (Söderman et al. 2001). Also, any “wait-and-see” approach results in underestimation of the number of revisions because those patients are not registered in the database. Even so, we believe that essential information is obtained from the assessment of implant survival with stem revision as endpoint, because this outcome can be regarded as an underestimation rather than overestimation of the problem.

It has been shown that restoration of the normal anatomical offset during THR is theoretically of advantage (Davey et al. 1993, McGrory et al. 1995). Unfortunately, we have no information on whether or not the normal anatomy after THR was restored with any particular offset in our analysis. We found that the Spectron stem and the Lubinus stem were associated with an increased risk of revision with increased offset and the use of a long neck. Interestingly, no such tendency was observed for the Exeter design. The reason for a seemingly increased sensitivity to high offset with use of a rough stem is unclear. It may be that a high offset facilitates debonding of the stem due to increased lever arm, resulting in particle production caused by abrasive wear between the stem and the cement mantle. In both the Lubinus SP2 and the Spectron EF Primary design, the head used to obtain maximum neck length has a skirt that might cause impingement and increased risk of dislocation.

It should be emphasized that some implant-related parameters may be biased by factors that are not known to us. The choice of level for neck resection may influence implant fixation. It would definitely influence the flexibility to change the amount of offset during surgery without causing pronounced changes in leg length and stem positioning, which would also influence the true offset. It may be that hips operated with maximum neck length represent technically difficult cases, and to some extent cases where the preoperative planning has been suboptimal.

It appears that there was a difference in outcome for the older version of the Exeter compared to the newer V40 design, as an increased incidence (although not statistically significant), was seen for aseptic loosening in the older version. On the other hand, a higher incidence of revision due to dislocation was seen for the V40 design (Table 5). However, this higher incidence of aseptic loosening for the older version of the Exeter stem must be interpreted with respect to the difference in follow-up time between the two designs, as revisions due to dislocation occur early and revision caused by aseptic loosening in general peaks even later than the mean follow-up for any of the subgroups in our study (Kärrholm and Herberts 2006).

With the evolution of new and more wear-resistant articulations, there is a trend to use larger heads, which can be expected to reduce the frequency of dislocation (Kelley et al. 1998, Berry et al. 2005, Conroy et al. 2008). There is a similar trend in Sweden, but the follow-up of the few patients is short. We therefore chose to exclude these cases since no relevant comparison could be made.

We found that the smallest sizes of Spectron and Lubinus implants represent an increased risk of stem loosening. The reason for the problems associated with small, unpolished cemented stems is not clear. These stems probably offer poor resistance against torsion, which means that they debond more easily and may then be a source of abrasive wear at the stem/cement interface. A polished stem will most likely cause less abrasion. Thus, if the femoral canal is narrow, polished stems may be preferred.

The overall performance of 3 of the most used stem designs during the past 8 years has been good. In 2006, these designs constituted about 71% of all stems inserted and their 10-year survival rate based on all reasons for revision varied between 93% and 96% (Kärrholm et al. 2008). In spite of this, 2 of the designs had implant variations associated with an increased risk of mechanical failure, which could probably have been avoided if the introduction of these variations had been done using more sophisticated techniques. Our findings underscore previous experiences from other implant designs, where changes in the surface finish or the addition of a new coating did not improve the clinical results (Rockborn et al. 1993, Middleton et al. 1998, Della Valle et al. 2005).
